# Removing Biases in Communication of Severity Assessments of Intimate Partner Violence: Model Development and Evaluation

**DOI:** 10.2196/43499

**Published:** 2023-04-28

**Authors:** Sverker Sikstrom, Mats Dahl, Emma Claesdotter-Knutsson

**Affiliations:** 1 Department of Psychology Lund Sweden; 2 Child and Adolescent Psychiatry Department of Clinical Sciences Lund Lund University Lund Sweden; 3 Department of Child and Adolescent Psychiatry Skåne University Hospital Lund Sweden

**Keywords:** debiasing, violence, natural language processing, machine learning, psychological, physical

## Abstract

**Background:**

To support a victim of violence and establish the correct penalty for the perpetrator, it is crucial to correctly evaluate and communicate the severity of the violence. Recent data have shown these communications to be biased. However, computational language models provide opportunities for automated evaluation of the severity to mitigate the biases.

**Objective:**

We investigated whether these biases can be removed with computational algorithms trained to measure the severity of violence described.

**Methods:**

In phase 1 (P1), participants (N=71) were instructed to write some text and type 5 keywords describing an event where they experienced physical violence and 1 keyword describing an event where they experienced psychological violence in an intimate partner relationship. They were also asked to rate the severity. In phase 2 (P2), another set of participants (N=40) read the texts and rated them for severity of violence on the same scale as in P1. We also quantified the text data to word embeddings. Machine learning was used to train a model to predict the severity ratings.

**Results:**

For physical violence, there was a greater accuracy bias for humans (r^2^=0.22) compared to the computational model (r^2^=0.31; *t*_38_=–2.37, *P*=.023). For psychological violence, the accuracy bias was greater for humans (r^2^=0.058) than for the computational model (r^2^=0.35; *t*_38_=–14.58, *P*<.001). Participants in P1 experienced psychological violence as more severe (mean 6.46, SD 1.69) than participants rating the same events in P2 (mean 5.84, SD 2.80; *t*_86_=–2.22, *P*=.029<.05), whereas no calibration bias was found for the computational model (*t*_134_=1.30, *P*=.195). However, no calibration bias was found for physical violence for humans between P1 (mean 6.59, SD 1.81) and P2 (mean 7.54, SD 2.62; *t*_86_=1.32, *P*=.19) or for the computational model (*t*_134_=0.62, *P*=.534). There was no difference in the severity ratings between psychological and physical violence in P1. However, the bias (ie, the ratings in P2 minus the ratings in P1) was highly negatively correlated with the severity ratings in P1 (r^2^=0.29) and in P2 (r^2^=0.37), whereas the ratings in P1 and P2 were somewhat less correlated (r^2^=0.11) using the psychological and physical data combined.

**Conclusions:**

The results show that the computational model mitigates accuracy bias and removes calibration biases. These results suggest that computational models can be used for debiasing the severity evaluations of violence. These findings may have application in a legal context, prioritizing resources in society and how violent events are presented in the media.

## Introduction

### Background

Social workers and decision makers within the legal system are often faced with extremely challenging decisions. This is in large part due to the complex and contested nature of the information the decisions are based on and also inherited cognitive biases of the decision maker. Nonetheless, society expects the decisions to be consistent, reliable, and fully justified, and correct evaluations of violence are of course crucial. These types of unwanted biases are shown to affect extremely crucial decisions, for example, permanency decisions in childcare [1,2], asylum adjudication [3], and parole decisions [4]. The evaluation of the severity of violence has also important implications for society in a general sense—how laws are instituted and applied and how resources are allocated to jurisdictions, the police, and social workers. Considering this, it is of utmost importance to identify and mitigate biases connected to these decisions.

Intimate partner violence (IPV) has been shown to be an underestimated problem, causing serious health issues for both men and women [5-8], as well as a large economical cost for individuals, families, and communities [9,10]. The World Health Organization (WHO) [11] defines IPV as “any behavior within an intimate relationship that causes physical, psychological or sexual harm to those in the relationship,” where the intimate partner can be anyone from a dating partner to a spouse. According to the Centers for Disease Control and Prevention [12], 25% of women and 10% of men in the United States have experienced some form of IPV. Even higher numbers have been shown by WHO [11] where the lifetime prevalence in the United Kingdom for sexual abuse is 16%, for physical violence is 25%, and for psychological violence is 34%.

Physical violence is considered any form of hitting, slapping, kicking, etc. The most studied violence is men’s physical violence against women [13]. Women also experience more severe forms of violence [14] and more overlapping forms of violence [15]. However, when including mild physical violence, other studies have found no sex difference [16]. It also seems like the time frame is important when looking at gender and IPV. Estimates are similar in women and men, but earlier-in-life estimated IPV is higher in women, making the time frame important when looking at IPV reports [16].

Less research has been conducted on psychological violence. A concern related to psychological violence is the lack of consensus of its definition [17]. Commonly, psychological violence includes intimidation, isolation, verbal attacks, victim blaming, and control of daily activities [6,12,18]. Although harder to define, psychological violence may have more serious consequences than physical violence, leading to increased incidences of depression, anxiety, posttraumatic stress disorder, and suicide; increased risk of cardiovascular disease; and premature mortality [19]. According to Lawrence et al [20], 8% of married partners engage in moderately severe psychological violence.

Comparison between psychological and physical violence is complex and depends on the role of the evaluators. Several studies show that third-party perceivers view physical aggression to be more harmful than psychological aggression [6,21,22]. However, when asking the victims, the opposite patterns have been found, and Follingstad et al [23] found that 75% of female victims found psychological violence to be worse than physical violence.

### Biases

Biases are various forms of cognitive mistakes made by the brain, often due to its rule-based processing of information but also external factors, such as time pressure, insufficient or ambiguous information, or too much or complex information. There are a great number of biases identified (eg, probability judgements [24], impression formation [25,26], primacy effect [27]), and most of them are automatic and unintentional and affect both the initial evaluation of information and adjustments performed later in the process. As mentioned before, these biases affect decisions of uttermost importance, such as permanency decisions in childcare [1,2] and asylum adjudication [3,4].

**Figure 1:**
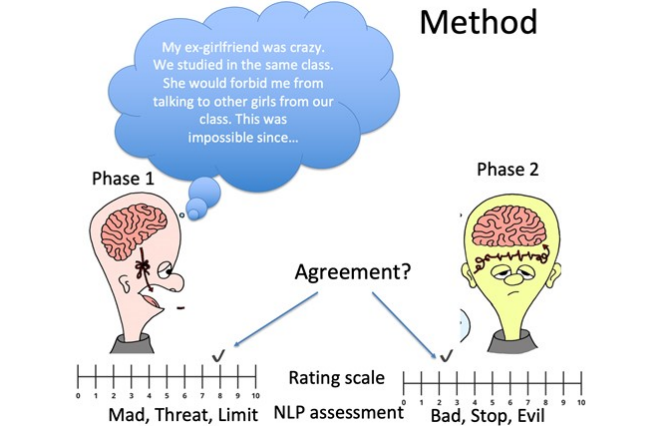
Communication of violence in P1 and P2. The self-experienced narratives of violence were written in P1 that were read by participants in P2. Participants in both phases rated the severity of violence and summarized the event in 5 descriptive words. NLP: natural language processing; P1: phase 1; P2: phase 2.

**Figure 2:**
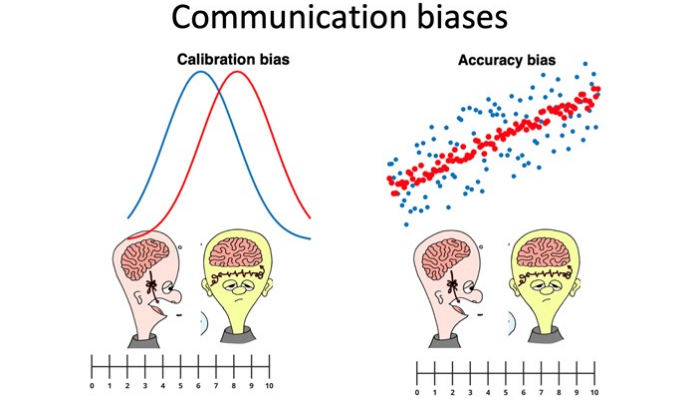
Calibration and accuracy biases. The calibration bias refers to the mean difference in the assessment of severity violence between P1 (blue) and P2 (red). The accuracy bias is about how well the assessments correlates between P1 (x axis) and P2 (y axis), where the red dots correspond to high accuracy and blue to low accuracy. P1: phase 1; P2: phase 2.

Most studies within the field have been conducted using vignettes, or video clips, of violent events [28] without the possibility of directly comparing real-life experienced and communicated violent events. In a previous study by our research group, we suggested a method where a group of participants were asked to describe self-experienced psychological and physical violent events and rate the severity of the violence [29]. The texts were subsequently read and evaluated by another set of participants. This method allowed us to directly compare the severity ratings of self-experienced and communicated events of violence, where the differences between these ratings were communication biases (Figure 1). In our study [29], we investigated 2 types of assessments (*calibration bias* and *accuracy bias*) under 2 types of violence (physical and psychological; Figure 2). By calibration bias, we mean the empirical phenomena by which an individual who experiences a violent event evaluates the severity of the violence of the event differently than a person, or an algorithm, to whom this event is communicated. This calibration bias can be measured on single events or averaged over a set of violent events. We found *calibration biases*, where psychological violence events were rated as more severe by participants experiencing them than by those reading about them, whereas this opposite pattern emerged for physical violence. By accuracy bias, we mean the correlation of the severity rating between pairwise evaluations of individuals who experience violent events and individuals, or algorithms, to whom these events have been communicated. Thus, several events are required to calculate an accuracy bias (as correlation cannot be conducted on single events). We found *accuracy biases* where the psychological violence was more difficult to communicate (ie, a lower correlation) than physical violence. Note that these biases are not necessarily dependent on each other, where it would be possible to have a calibration bias but not an accuracy bias, or vice versa, on the same set of events. These biases may have severe implications for both victims and offenders, as they indicate that evaluators have poor insight into how the events are experienced by the victims.

### Biases or Perceptual Differences

Perceptual differences generally arise from the diversity of how information is processed by different individuals. There are individual differences in the interpretation and evaluation of information that naturally generate differences in assessment. It could be argued that these differences are merely the results of evaluations based on different “subjective realities” and therefore not “biases” as traditionally defined—a systematic deviation from a normatively defined pattern. However, in this study, the focus is not on exploring the origin of the discrepancies in evaluation but on the effect of the differences. The key issue here is whether the victim and the evaluator have the same opinion about the intensity of the violence. That is, in this particular context of communication of the severity of violence, it is vital to remove, or at least mitigate, the discrepancies, or biases, between the sender and the receiver in order to correctly assess the severity of the violence. In fact, precise communication of the seriousness of violence in intimate relationships is essential for the proper assessment of perpetrators and victims in contexts such as court proceedings, custody cases, and relationship continuity. We have chosen to use the term “bias” when describing the various forms of differences found in the evaluations rather than simply perceptual differences. This is because there are, undoubtedly, elements of both naturally occurring subjective differences and systematic deviations from a normative pattern that constitutes the differences in evaluation.

### Computational Language Models and Rating Scales

Computational models of semantic representation can be obtained by looking at the context in which words are represented, where words with similar meaning tend to be placed in related contexts. A computational model of noncontextual semantic representations is latent semantic analysis (LSA) [30] that uses information of word frequency co-occurrences in texts. In this model, a co-occurrence matrix from a corpus is first generated, and then a data compression algorithm is used that maintains as much information as possible in a smaller number of dimensions called word embeddings. More recent contextual embedding models, for example, Bidirectional Encoder Representations from Transformers (BERT) [31], also allow an understanding of the grammatical structures of texts and are based on neural networks that are trained with deep learning in combination with transformers that implement attention mechanisms where different parts of the texts are attended to. Although contextual models typically perform better than noncontextual models in tasks that requires contextual knowledge, contextual models (ie, BERT) have been shown to have similar performance as noncontextual models (ie, Word2vec) in noncontextual tasks [32]. In this paper, we used noncontextual grammar-free data where participants described violence with keywords, and therefore, we chose the LSA model.

Recent studies have shown that language-based responses from directed questions can be used to predict rating scales with reasonable high accuracy. For example, Kjell et al [33] let participants generate keywords, and commonly used ratings scales, related to mental health (ie, depression, anxiety, harmony, and certification). By using natural language processing (NLP, ie, LSA) to quantify the meaning of the words to a vector, and machine learning (ie, multiple linear regression) to train the vector to predict the rating scales, they showed that these computational methods could predict the rating scales well. Later work on satisfaction and harmony, that combined several types of response formats and constructs, showed high correlation to rating scales (r^2^=0.72), which challenges the theoretical limits of ratings scales, as measured by test-retest scores or interitem reliability [34]. However, to the best of our knowledge similar models have not been applied to measure the severity of violence.

The aim of this study is to investigate whether computational language models can be used to remove accuracy and calibration biases in severity ratings between experienced and communicated narratives of psychological and physical IPV. To the best of our knowledge, this has not been studied in the previous literature. Our hypothesis is that computational language models can remove these biases and make the ratings more accurate.

## Methods

### Data

We studied the data set collected by Sikström et al [29], where accuracy and calibration biases were found for psychological and physical violence.

The data analyzed in this study [29] were collected in 2 phases (phase 1 [P1] and phase 2 [P2]) using the Prolific Academic website for online recruiting. The process used is described next. For a more detailed description, see Sikström et al [29].

### Participants

The participants were recruited using an ad presenting the study on the Prolific Academic website. The inclusion criteria were (1) being a heterosexual US citizen, (2) aged 18 years or older, (3) speaking English as the native language, and (4) having had at least 1 relationship lasting for 6 months. All those who fulfilled the inclusion criteria and completed writing the texts and making the evaluations were included in the samples. In P1, the sample consisted of 71 participants (n=22, 31%, females and n=49, 69%, males). The mean age was 34.5 (SD 11.9) years. In P2, the sample consisted of 240 participants (n=170, 50%, females and n=170, 50%, males). The mean age was 35.0 (SD 12.2) years.

### Procedure

In P1, participants (N=71) were instructed to write a text paragraph and type 5 keywords describing an event where they experienced physical violence and 1 keyword describing an event where they experienced psychological violence in an intimate partner relationship. They were also asked to rate the severity of the violence from 0 (not serious at all) to 10 (very serious). In P2, another set of participants (N=40) read the texts generated in P1 and rated them for severity of violence on the same scale as in P1. Half of the participants rated the texts related to psychological violence and the other half the texts related to physical violence, so the total number of rated texts in P2 was 40 × 68 = 2720. They also described the severity of the read events using 5 descriptive keywords. For details of the study, see Sikström et al [29].

### Overview of Data Processing

We first quantified the text data to word embeddings (ie, a vector describing the meaning of a text) and then used machine learning to map the embeddings to a scale of severity of violence. The mapping of words to word embeddings was conducted using a version of LSA [30], and then we used multiple linear regression to map the embeddings to severity ratings. This method is described in detail by Kjell et al [33], and here we provide a brief overview of the algorithm. The analysis was conducted using an online platform for statistical analysis of semantic representations called SemanticExcel, which is developed by the author of this paper [35].

### Creation of Word Embeddings

The Google N-gram (N=5) database [36] was used as input. This database was chosen as it is perhaps the largest collection of N-grams publicly available for the English language, consisting of terabytes of text data. We chose to create the co-occurrence matrix on 5-grams, as this provided an opportunity of a larger ±4 window that is beneficial for semantic analogy tasks, whereas smaller windows based on bigrams show good task performance on syntactic analogies [37]. This window size has been successful in previous publications [35]. For a more elaborated investigation of how the window size and other factors influence accuracy in different tests, see the Global Vectors for Word Representation model by Pennington et al [37]. Based on this, a word-by-word co-occurrence matrix was generated with 120,000 words with the most frequency on the rows and similarly for 50,000 columns. Thus, each cell represented the number of times that 2 words co-occurred in the 5-grams in Google’s data set. To attenuate frequency artifacts, each cell was normalized with log(co-occurrence frequency + 1). This co-occurrence matrix was reduced using a data compression algorithm called singular value decomposition (SVD) that maintains as much information as possible on as few dimensions as possible (ie, the first dimensions carry the most information), where 512 dimensions were maintained. SVD is similar to principal component analysis (PCA) more commonly used in the psychological literature; however, the difference is that the SVD matrix does not need to be centered and is not necessarily truncated. The length of each vector, representing a word embedding, was normalized to 1. The 5 words generated by the participants were summarized to 1 embedding by adding the embedding for each word and normalizing the length of the vector to 1.

### Creating and Applying a Model for the Severity of Violence

Machine learning was used to train a model to predict severity ratings. With training, we mean that machine learning adopts parameters based on the input data (ie, words represented as semantic vectors) to estimate the severity of violence ratings. The P1 data contained too few data points (N=136) to create a machine learning model to predict the severity of violence ratings based on the word with a high accuracy. Instead, we created a model on the words and severity scale generated in P2 (N=2720). Although it would have been possible to create the model on data from both P1 and P2, we wanted to have a pure model that only had access to the information in P2, not P1. Furthermore, the number of data points in P1 were so few in comparison to P2 that we argued that adding the P1 data would not make a significant contribution.

Thus, the model was based only on the keywords and not on the free text data. The reason for this was that the keywords were found to be more informative than the rating scales, so adding the free text data (from P1) did not improve the accuracy over and beyond just using the keywords. Furthermore, the participants in P2 did not generate free text, so it would not have been possible to train on the P2 data.

Standard (ie, we did not use Lasso regression) multiple linear regression (y = c × x), using the word embeddings from P2 as input (x), was used to predict the associated severity ratings (y) by adapting the coefficients (c). For the predictions trained to binary outcome values (eg, physical versus psychological violence), multiple logistic regression was used. The model was evaluated using a standard leave-out cross-validation procedure, where the model was trained on 90% of the data and evaluated on the left-out 10% of the data. The partitions were created by 1 unique partition, so the same partitions were used during each training and all data points were evaluated (ie, resampling was not conducted). The number of dimensions used was optimized to a mean value of 197 (SD 33) first dimensions (ie, the higher dimensions were not used) using the training data set in each cross-validation fold and where we applied the model that generated the highest fit to the data on the test data set. The fit of the model was measured using Pearson correlation (r) between the predicted value and the empirical value of severity ratings using the described leave-out cross-validation method and was found to be r^2^=0.37. The leave-out groups were based on the text that was generated in P1, so texts that the model was trained on were never used during testing. The model generated from the P2 data was applied to the P1 data, so predicted values were obtained for each of them.

### Word Clouds

The model described before was applied to create word clouds (Multimedia Appendices 1-3). In Multimedia Appendix 1, the model was trained on a binary value where texts describing physical violence were coded as 1 and texts describing physical violence were coded as 0. In Multimedia Appendix 2, the model was trained to predict the calibration bias (ie, the ratings in P2 minus the ratings in P1). In Multimedia Appendix 3, the model was trained to predict the severity ratings. Each word was measured using the model described before. The color coding of each word represents the word’s z-value and the font size the frequency of the word in the data set. Words more centrally located in the word clouds have stronger effect sizes than those that are peripheral.

### Ethical Considerations

The study was approved by the Swedish Ethical Review Authority (Dnr 2022-06518-01).

## Results

### Biases

**Figure 3:**
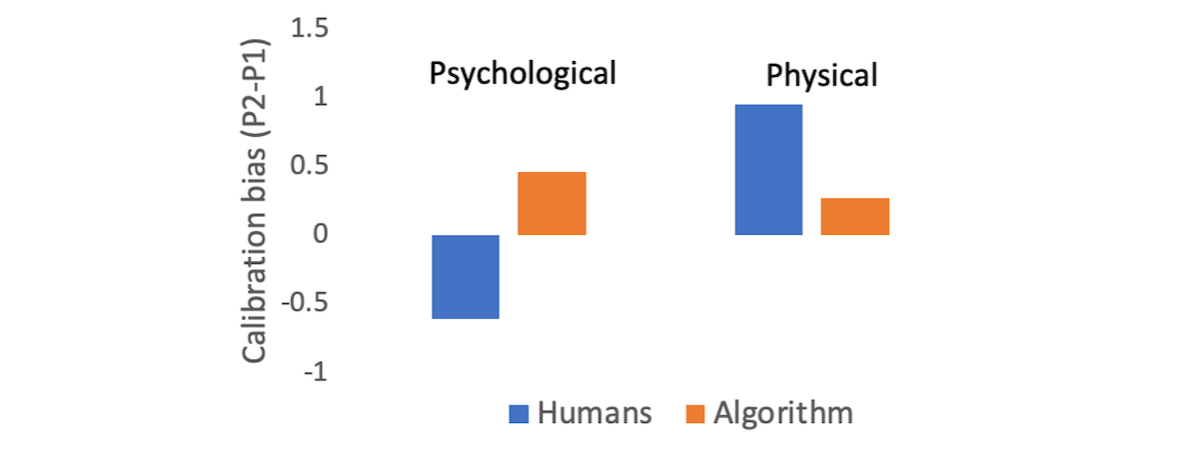
Calibration bias for humans and the algorithm. The y axis shows the calibration bias (ie, the severity of violence in P2 minus the severity of violence in P1) for human ratings (blue) and the model estimate of the ratings (red). The two leftmost bars show the results for psychological violence and the two rightmost bars for physical violence. P1: phase 1; P2: phase 2.

**Figure 4:**
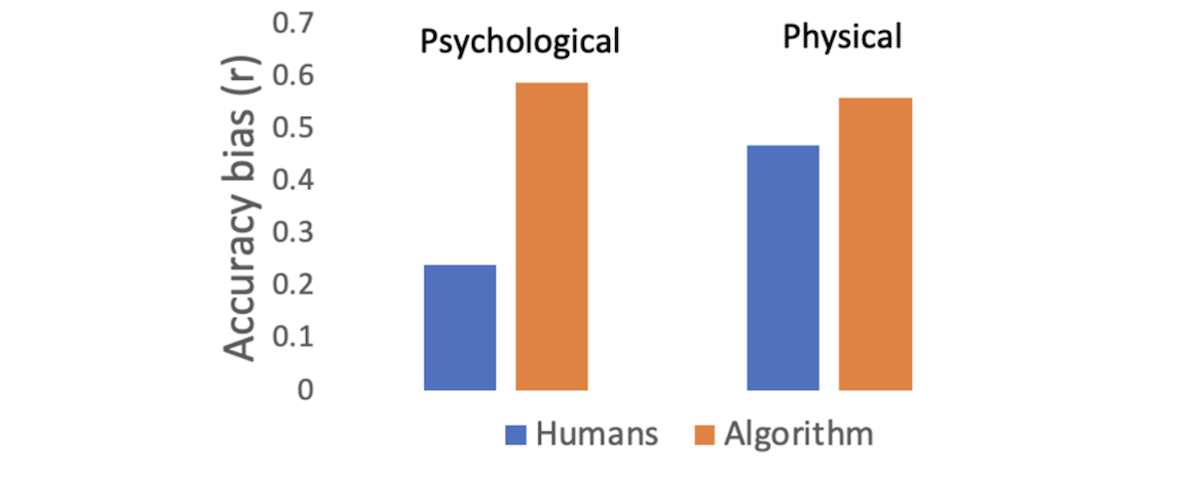
Accuracy bias for humans and the algorithm. The y axis shows the accuracy bias (ie, Pearson correlation between the severity of violence in P1 and P2) for human ratings (blue) and the model estimate of the ratings (red). The two leftmost bars show the results for psychological violence and the two rightmost bars for physical violence. P1: phase 1; P2: phase 2.

The calibration bias was measured by subtracting the mean value for P1 from the mean value for P2 (for humans) and the predicted values for P2 (the algorithm); see Figure 3. The accuracy bias was measured with Pearson correlation between the P1 and P2 ratings (for humans) or the predicted value severity ratings (for the algorithm); see Figure 4. Furthermore, these biases were calculated for the whole data set, the psychological condition, and the physical condition (Table 1).

The *accuracy bias*, or how accurately the experienced severity of violence in P1 was evaluated by participants reading the texts in P2, was measured using Pearson correlation between participants in P1 and P2. A Pearson correlation score (r) was calculated for each participant using their human severity rating in P2 and the severity ratings for the same event by participants in P1. These Pearson correlations were also calculated using the model predictions instead of the human ratings in P2. This was performed separately for each participant in P2, and 2-sided *t* tests were conducted for testing whether r values differed between humans and the computational model. For physical violence, there was a greater accuracy bias (ie, lower r) for humans (r^2^=0.22) compared to the computational model (r^2^=0.31; *t*_38_=–2.37, *P*=.023). Similarly, for psychological violence, the accuracy bias was greater for humans (r^2^=0.058) than for the computational model (r^2^=0.35; *t*_38_=–14.58, *P*<.001).

For *psychological violence*, a calibration bias was found for humans but not for the computational model. Participants in P1 experienced psychological violence as more severe (mean 6.46, SD 1.69) than participants rating the same events in P2 (mean 5.84, SD 2.80; *t*_86_=–2.22, *P*=.029<.05), whereas no calibration bias was found for the computational model (*t*_134_=1.30, *P*=.195). However, no calibration bias was found for physical violence for humans between P1 (mean 6.59, SD 1.81) and P2 (mean 7.54, SD 2.62; *t*_86_=1.32, *P*=.190) or for the computational model (*t*_134_=0.62, *P*=.534).

**Table 1:** Severity of violence as evaluated by humans and the computational model: correlations, means (SDs), and biases.

Condition	Data points, N	Correlation, r			Mean (SD)				Bias (difference in means)	
		Between P1^a^ and P2^b^	Between P1 and MP1^c^	Between P1 and MP2^d^	P1	P2	MP2	Human bias (P1 – P2)		Computational bias (P1 – MP2)
Both psychological and physical	2720	0.36	0.48	0.58	6.52 (1.75)	6.69 (2.84)	6.88 (2.67)	0.17		0.36
Psychological	1360	0.24	0.42	0.59	6.46 (1.69)	5.85 (2.80)	6.91 (2.31)	–0.61		0.46
Physical	1360	0.47	0.54	0.56	6.59 (1.81)	7.54 (2.62)	6.85 (2.99)	0.95		0.27

^a^P1: phase 1.

^b^P2: phase 2.

^c^MP1: predicted value of P1.

^d^MP2: predicted value of P2.

### Indicative Words

Word clouds indicative of calibration biases, the severity of violence, and the type of violence were generated based on the semantic scales created with the multiple linear regression training described before.

Multimedia Appendix 1 shows word clouds indicative of psychological violence (left) and physical violence (right); r^2^=0.04, *P*<.001. Physical violence was indicated with words such as *abusive*, *violent*, *and physical*, whereas psychological violence was associated with words such as *mean*, *controlling*, and *manipulative*.

Multimedia Appendix 2 shows word clouds related to negative calibration bias (left) and positive calibration bias (right); r^2^=0.10, *P*<.001 (Figure 4). For example, *physical*, *abusive*, and *violent* are words indicative of a positive calibration bias (Multimedia Appendix 1, left panel), whereas *psychological*, *mild*, and *verbal* are words indicative of a negative calibration bias (Multimedia Appendix 1, right panel). Visual inspection showed that words predicting a positive calibration bias of violence also tended to predict words associated with physical violence or high severity ratings, whereas words that predicted a negative calibration bias were associated with psychological violence and low severity ratings. This relationship was confirmed by creating a model for predicting the calibration score (r^2^=0.07, *P*<.001) and a model for predicting the physical versus the psychological condition (r^2^=0.17, *P*<.001). The results showed a positive Pearson correlation between the predicted calibration bias and the predicted severity ratings (r^2^=0.61) and between the predicted calibration bias and the predicted physical condition (r^2^=0.16).

Multimedia Appendix 3 shows word clouds related to low (left) and high (right) severity scores (r^2^=0.41, *P*<.001). For example, *emotional*, *psychological*, and *sad* are words indicative of low severity scores, whereas *violent*, *abusive*, and *dangerous* are words indicative of high severity scores. A visual inspection of the word clouds showed that low severity scores are related to words also describing psychological violence, whereas high severity scores are related to words also describing physical violence. This finding was confirmed by a positive Pearson correlation between the predicted severity scores and the physical condition (r^2^=0.24) and a positive Pearson correlation between the predicted severity score and the predicted calibration bias (r^2^=0.61).

### Comparing Bias, Physical, and Psychological Data

There was no difference in the severity ratings between psychological and physical violence in P1. However, the bias (ie, the ratings in P2 minus the ratings in P1) was highly negatively correlated with the severity ratings in P1 (r^2^=0.29) and in P2 (r^2^=0.37), whereas the ratings in P1 and P2 were somewhat less correlated (r^2^=0.11) using the psychological and physical data combined.

## Discussion

### Principal Findings

The aim of this study was to determine whether computational language models can be used to remove accuracy and calibration biases in severity ratings between experienced and communicated narratives of psychological and physical IPV. We used a data set collected by Sikström et al [29]. The calibration biases found in humans were not found in the computational model. Furthermore, the accuracy in predicting the severity of psychological and physical violence was higher for the computational model compared to humans.

Previous data have shown that psychological violence is considered by those exposed to be more severe than physical violence [19,23]. Our data showed no such difference for humans or the computational model.

We previously investigated the data set regarding differences in the perception of the severity of psychological and physical violence [29]. Our results showed that the confidence of such severity ratings needs to be adjusted for several factors, such as whether it is self-experienced or communicated, the type of violence, and the gender of the victims and raters [29]. In this paper, we showed that these biases can be debiased using computational models.

Using computational models to debias language-based data on violence may have important practical implications. It may increase legal certainty by improving the correct assessment in the estimation of victims’ suffering and may aid in determining to what extent they should be compensated for the consequences of the violence. It may also provide a fairer trial to perpetrators, given that the punishment can be better connected to the severity of the crime. Furthermore, increased knowledge and studies regarding measuring the severity of psychological and physical violence may influence political decisions by improving how society allocates resources that deal with these issues, for example, to courts, the police, social workers, help lines, and health institutions. These results suggest that the computational model can mitigate or even remove biases found in humans. To the best of our knowledge, our study is the first of its kind. The results found here may be improved in future studies using larger data sets, asking participants more elaborate questions, and fine-tuning the computational algorithms.

Another finding, as is evident from the word clouds and supporting analysis, is that words indicative of severe violence also predict both word responses showing a positive calibration bias and word responses indicating physical violence. Thus, physical violence with a positive calibration bias is related to words describing severe violence. In contrast, low severity of violence is indicative of negative calibration biases and psychological violence.

A concern of this study is that the severity rating in P1 may not be a proper measure of how severe the violence is. In our view, we do not see this as a limitation, as we are purely interested in measuring the differences in the severity ratings in P1 compared to P2. Thus, our approach does not take a strong stand regarding the extent to which the P1 rating truly reflects an objective grounded truth of the severity of violence. The proposed machine learning method could also be used for training the data to other severity measures. Future studies may investigate how well our method can handle other severity measures.

### Limitations

This study has several limitations. First, our approach does not always consider the possibility that the rating scales may be uniquely interpreted by the participants, so a 7 on the severity scales for one participant may be interpreted as 5 by another participant. However, a unique aspect of using language as an outcome variable is that it allows participants to freely express the unique aspects of how they perceive violence, whereas rating scales do not allow unique expressions. This suggests that language data provides better opportunities for person-centered evaluations and a unique description of victims’ view of violence compared to rating scales. The second limitation of the study is that our measure of accuracy bias is based on within-participant correlations, making it less sensitive to differences in how different participants experience the scale.

The third limitation is that the studied communication is limited to texts. Thus, in comparison to real-life scenarios, it was not possible to create follow up questions. Self-reported data could be limited by recall bias—hence the limited generalizability [38].

The fourth limitation of the study is that the participants may have had a lower incentive to make an effort to communicate the violent events carefully compared to real-life settings where the stakes are higher. However, at the same time, they also had less incentive to make false statements. Another difference from real-life settings is that communication of violence is typically made in spoken, not written, form and that rating scales are rarely used.

The fifth limitation of the study is that the generalizability of the result is limited as the respondents were limited to Prolific Academic users, who may have limited experience of severe violence or where significant time may have elapsed since these experiences. To strengthen the results, future studies should use a more diverse sample, for example, including participants who have recently experienced severe violence. The proposed method debiasing the severity of violence could potentially be applied for removing bias connected to various groups based on race, gender, age, culture, etc.

The sixth limitation is the scale used for severity measurement. This scale was chosen as it directly asks for the rater’s subjective experience of the severity of violence that we also aimed to measure. Other scales for measuring violence, for example, the Conflict and Tactic Scale (CTS-2) [39] or the Index of Wife Abuse (ISA) [40], may have a goal to measure the severity of violence more objectively; however, this would not fulfill our purpose of measuring subjective biases in severity ratings.

### Implications

This study has important implications for victims, offenders, and the society around them that evaluates the severity of violence. Computational support for evaluating the severity of violence may improve legal justice, leading to better aid for victims and more proper treatment of offenders.

### Conclusion

Our analysis supports the idea that computational language models can mitigate or remove bias in communication of violence in texts that otherwise is found in humans.

## Appendix

Multimedia Appendix 1 Psychological violence (left) and physical violence (right); r^2^=0.04, *P*<.001. Words indicative of psychological violence are shown on the left and physical violence on the right. The color coding represents z-values, and the font size the frequency of the words in the data set.

Multimedia Appendix 2 Negative calibration bias (left) and positive calibration bias (right); r^2^=0.10. The word clouds show words most discriminative for negative calibration bias (left) and high calibration bias (right). The word embeddings predicted the calibration bias (ie, the ratings in P2 minus the ratings in P1) with reasonably high accuracy (r^2^=0.10, *P*<.001). The right word cloud shows words that predict positive calibration bias (ie, higher ratings in P2 compared to P1), whereas the right world cloud shows words predicting negative calibration bias.

Multimedia Appendix 3 Low severity ratings (left) and high severity ratings (right); r^2^=0.34. Words indicative of psychological violence are shown on the left and physical violence on the right. The color coding represents z-values, and the font size the frequency of the words in the data set.

## Abbreviations

BERT: Bidirectional Encoder Representations from Transformers
IPV: intimate partner violence
LSA: latent semantic analysis
NLP: natural language processing
P1: phase 1
P2: phase 2
SVD: singular value decomposition
WHO: World Health Organization

## Data Availability

The data set is available from the author SS upon request.
