# Supplementary figures and images for: Removing Biases in Communication of Severity Assessments of Intimate Partner Violence: Model Development and Evaluation
Source: J Med Internet Res. 2023 Apr 28;25:e43499. doi: 10.2196/43499 (PMC10182463; doi:10.2196/43499)

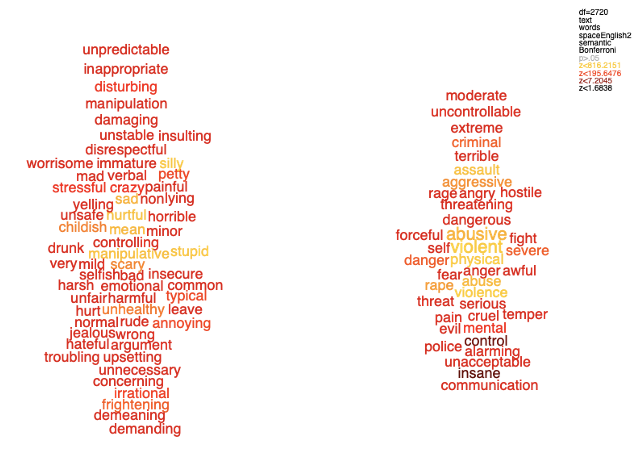

Supplement: Multimedia Appendix 1 [file jmir_v25i1e43499_app1.png]

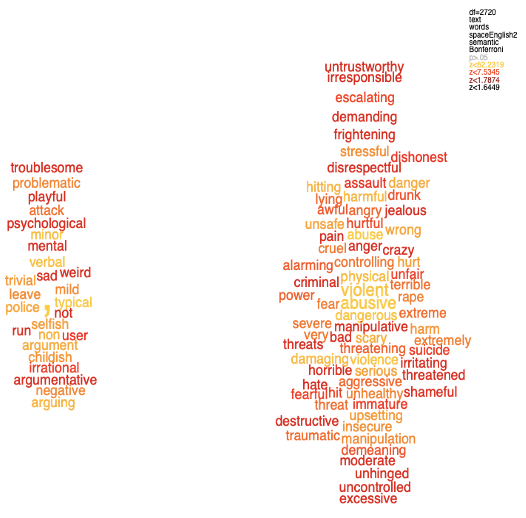

Supplement: Multimedia Appendix 2 [file jmir_v25i1e43499_app2.png]

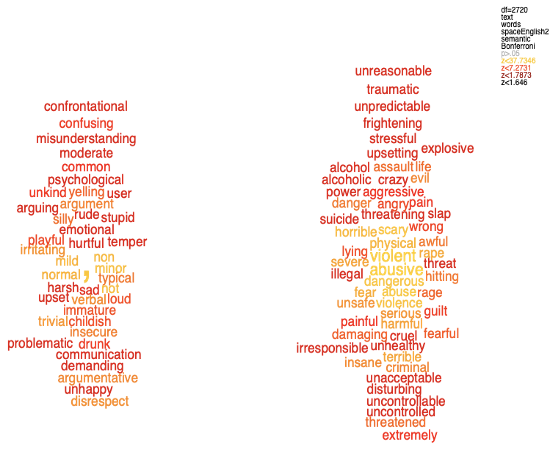

Supplement: Multimedia Appendix 3 [file jmir_v25i1e43499_app3.png]
